# Supplementary material for: A bead-based GPCR phosphorylation immunoassay for high-throughput ligand profiling and GRK inhibitor screening
Source: Commun Biol. 2022 Nov 9;5:1206. doi: 10.1038/s42003-022-04135-9 (PMC9646841; doi:10.1038/s42003-022-04135-9)
Supplement: Supplementary file 3 — Description of Additional Supplementary Files [file 42003_2022_4135_MOESM3_ESM.pdf]

Description of Additional Supplementary Files

File name: Supplementary Data 1

Description: Source assay data behind the graphs in the main manuscript (7TM phosphorylation assays, Arrestin and GRK binding assays).

File name: Supplementary Data 2

Description: Source assay data behind the graphs in the main manuscript (GIRK channel assays).

File name: Supplementary Data 3

Description: Source assay data behind the graphs in the supplementary information.
